# Supercritical CO_2_ extraction in chia oils production: impact of process duration and co-solvent addition

**DOI:** 10.1007/s10068-018-0316-2

**Published:** 2018-01-17

**Authors:** Grzegorz Dąbrowski, Iwona Konopka, Sylwester Czaplicki

**Affiliations:** Chair of Plant Food Chemistry and Processing, Faculty of Food Sciences, University of Warmia and Mazury in Olsztyn, Pl. Cieszyński 1, 10-726 Olsztyn, Poland

**Keywords:** Chia oil, Supercritical extraction, Extraction time, Acetone addition, Bioactive compounds, Fatty acids

## Abstract

This study was conducted to show impact of supercritical fluid extraction using pure SC-CO_2_ or SC-CO_2_ enriched by 2, 6, and 10% of acetone, simultaneously varied by the extraction time (1 vs. 5 h), on the quality of chia seed oils. Obtained oils were relatively similar in the fatty acid composition, but they differed in total content of phytochemicals (from 4956 to 6391 mg/kg of oil). Among them, three oils were the most different: oil extracted 1 h with pure SC-CO_2_ (the most abundant in squalene, sterols, and tocopherols), oil extracted 5 h with pure SC-CO_2_ (the poorest in squalene, polyphenols, and carotenoids, with medium level of sterols and tocopherols) and oil extracted 1 h with SC-CO_2_ enriched by 10% acetone (the most abundant in polyphenols and carotenoids). Such unique chia oils can be valuable for special market needs, such as cosmetics, pharmaceuticals, functional food or diet supplement production.

## Introduction

In recent years, there has been a growing market of unique plant oils rich in health-promoting unsaturated fatty acids and abundant in various classes of terpenoids. A crucial problem during the production and storage of such oils is their high susceptibility to oxidation. An example of this type of oil is *Salvia hispanica* L. (chia) seed oil, which is one of the richest plant sources of n-3 α-linolenic acid. In this oil, n-3 α-linolenic acid accounts for approx. 60–67% of total fatty acids [1–3]. Chia oil also contains high amounts of tocopherols and phytosterols, accompanied by lower amounts of squalene and carotenoids [4]. The content and profile of phytochemicals, as well as the shelf life of chia oil, is highly related to the method of its extraction. Our previous study [4] showed that the classical extraction with acetone instead of *n*-hexane improved oil recovery, increased the content of phytochemicals and, in summary, significantly affected the chia oil induction period. Oxidative oil stability was significantly correlated with the content of carotenoids (r = 0.96) and phenolic compounds (r = 0.99).

In the case of industrial oil extraction, the process is limited by oil recovery, impact on oil nutritional value and satisfactory shelf life of the obtained product. The most commonly used plant oil extraction methods are pressing, extraction with organic solvents or a combination of these processes. Solvent extraction is the most efficient method and allows the recovery of over 98% of available oil. The disadvantages of this process are high costs and the necessity of using highly inflammable chemicals and possible solvent residues in the obtained oil [5]. Screw pressing is less expensive than solvent extraction because it requires only a mechanical power input instead of heat or organic solvents [6]. Pressing allows the recovery of 86–92% of oil when a two-stage process is applied [7]. Oil recovery in pressing is dependent on several factors, i.e. cracking, de-hulling, moisture content or heat conditioning of seeds [4, 8].

A novel technique that can avoid the above difficulties is supercritical fluid extraction (SFE). SFE is environmentally friendly because it does not require inflammable organic solvents. The most commonly used supercritical solvent is CO_2_ which is safe, non-toxic and easily available at a high purity level and low price [9]. Supercritical CO_2_ is a non-polar, effective solvent for plant lipids. A good way of improving the supercritical carbon dioxide (SC-CO_2_) extraction grade of polar or amphiphilic compounds is the addition of modifiers which can increase the solvating power. More polar modifiers significantly increase the solubility of polar compounds (e.g. polyphenols) [10]. This effect comes from two main sources. Firstly, a modifier can increase the mass transfer by cleaving bonds between plant matrix and solute [11]. The second mechanism of improving the extractivity of polar compounds consists of better penetration of SC-CO_2_ into the matrix, due to swelling of the sample matrix with a co-solvent. A modifier also produces an analyte-co-solvent interaction that increases the solubility of the analytes in SC-CO_2_ [12]. In most cases, the modifiers in SC-CO_2_ extraction are alcohols (ethanol and methanol, respectively). Other considered co-solvents are water, acetone, isopropanol, dichloromethane, *n*-hexane and mixtures of solvents [13, 14]. Another important factor influencing the yield and composition of extract in the SFE process is the extraction time or SC-CO_2_/sample mass ratio. There are many studies which say that extract mass increase is the fastest at the beginning of SFE process, when the value of the SC-CO_2_/sample mass ratio is low [15–18]. Some reports also concern the influence of extraction time on the content of selected phytochemicals in plant oils. According to de França et al. [15], the concentration of carotenoids in buriti (*Mauritia flexuosa*) oil constantly increases with the rise of used SC-CO_2_ amount. In contrast, squalene, tocopherol and phytosterol mass transfer is faster at the beginning of the process, which results in their higher concentration in oils extracted for a shorter time. Depending on the pressure, the highest quantity of squalene was extracted in the first 30 min of the SFE process (over 60% of its total content), while the highest levels of tocopherols and sterols were extracted in the first hour of SFE process [19, 20].

The lipid fraction of each plant material is a mixture of various compounds. They differ in chemical structure, polarity and localization in tissues and cells, which affects their susceptibility to extraction. Seed lipids are deposited almost exclusively in oil bodies built from a hydrophobic core containing triacylglycerols surrounded by a phospholipid monolayer stabilized by phytosterols and specific hydrophobic proteins [21]. Minor lipid parts are localized in other cell membranes and organelles and, in general, are less soluble and extractable. This indicates that unique plant oils, enriched in several phytochemicals, may be obtained by modifying the extraction parameters. The present study was conducted to detect the possibility of the production various types of chia oils using SC-CO_2_ extraction modified by using acetone as a solvent modifier and by changing the extraction time duration.

## Materials and methods

### Material

Commercial chia seeds were purchased from a local market in Olsztyn, Poland. The total lipid content was 34.8% (determined according to Folch et al. [22] procedure). Seeds were packed in plastic bags and stored at 5 ± 2 °C until use. Before extraction seeds were bring to room temperature and ground in a A20 laboratory mill (IKA-Werke, Staufen, Germany) to obtain particles below 800 µm.

### Supercritical carbon dioxide extraction

Extraction was carried out in a 50 mL stainless steel vessel with the use of an SFE extractor (JASCO International Co., LTD., Tokyo, Japan). The process took1 or 5 h and a SC-CO_2_ under a working pressure of 28 MPa at 70 °C and flow rate 8 mL/min was used. Extractor was coupled with the Hewlett-Packard 1050 series pump (Palo Alto, CA, USA) which was used to pump acetone (3 variants: 2, 6 and 10% (v/v) in main solvent stream). The residual acetone in oils was evaporated in rotary evaporator type R210 (Büchi Labortechnik AG, Postfach, Switzerland) at temperature 45 °C and pressure 5 kPa.

Oil recovery was calculated by the Eq. (1) in relation to result of Folch method (total lipids content):

1 $${\text{Oil}}\,{\text{recovery}}\, ( {\text{\%)}} = \frac{{\left[ {\frac{{{\text{weight}}\,{\text{of}}\,{\text{fat}}}}{{{\text{weight}}\,{\text{of}}\,{\text{sample}}}}} \right] \times 100\% }}{{{\text{total}}\,{\text{fat}}\,{\text{content}}\, ( {\text{\%)}}}} \times 100\%.$$

### Determination of fatty acids composition

Fatty acids methyl esters were analyzed by gas chromatography with mass spectrometry using a GC-MS QP2010 PLUS (Shimadzu, Kyoto, Japan) system according to the parameters described by Czaplicki et al. [23]. Separation was performed on a BPX70 (25 m × 0.22 mm × 0.25 µm) capillary column (SGE Analytical Science, Victoria, Australia) with helium as a carrier gas at a flow rate of 1.3 mL/min. The injector temperature was set at 230 °C and the column temperature was programmed as follows: a subsequent increase from 150 to 180 °C at the rate of 10 °C/min, to 185 °C at the rate of 1.5 °C/min, to 250 °C at the rate of 30 °C/min, and then 10 min hold. The GC-MS interface and ion source temperatures were set at 240 °C. The temperature of the was 240 °C and the electron energy 70 eV. The total ion current (TIC) mode was used in 50–500 m/z range.

### Determination of phytosterols and squalene

The content of sterols and squalene was determined by gas chromatography coupled with mass spectrometry (GC-MS QP2010 PLUS, Shimadzu, Kyoto, Japan) according to the method described by Czaplicki et al. [23]. Saponified and extracted compounds with internal standard (5α-cholestane, Sigma-Aldrich, Poznań Poland) were re-dissolved in 100 µL of pyridine and 100 µL BSTFA (N,O-bis (trimethylsilyl) trifluoroacetamide) with 1% TMCS (trimethylchlorosilane) and silylated at 60 °C in 60 min. Compounds were separated on ZB-5MSi (30 m × 0.25 mm × 0.25 µm) capillary column (Phenomenex Inc., Torrance, CA, USA) with helium as a carrier gas (0.9 mL/min). The injector temperature was set at 230 °C and the column temperature was programmed as follows: 70 °C for 2 min, a subsequent increase to 230 °C at the rate of 15 °C/min, to 310 °C at the rate of 3 °C/min, and then 10 min hold. The GC-MS interface and ion source temperatures were set at 240 and 220 °C, the electron energy 70 eV. The total ion current (TIC) mode (100–600 m/z range) was used. Compounds were identified on the basis of their mass spectra. Quantitative analysis of compounds was performed with the use of 5α-cholestane as an internal standard.

### Determination of tocopherols

Analysis of content of tocopherols was carried out with HPLC-FLD technique, according to the method described by Dąbrowski et al. [4]. The oil in *n*-hexane solution (1%, m/v) was injected into the chromatographic system. The analysis was performed using a 1200 series liquid chromatograph manufactured by Agilent Technologies (Palo Alto, CA, USA), equipped with a fluorescence detector. The separation was done on a LiChrospher Si 60 (250 mm × 4 mm, 5 µm) column (Merck, Darmstadt, Germany). A 0.7% isopropanol solution in *n*-hexane at a 1 mL/min flow rate was used as a mobile phase. The fluorescence detector was set at 296 nm for excitation and 330 nm for emission. Peaks were identified on the basis of retention times determined for α, β, γ and δ tocopherol standards (Merck, Darmstadt, Germany) separately, and their content was calculated using external calibration curves.

### Determination of carotenoids

Carotenoids were analysed with a RP-HPLC technique according to the method described by Czaplicki et al. [23]. Briefly, the analysis was carried out using a 1200 series liquid chromatograph manufactured by Agilent Technologies (Palo Alto, CA, USA), equipped with a diode array detector (DAD) from the same manufacturer. Separation was performed at 30 °C on a YMC-C30 150 × 4.6 mm, 3 µm column (YMC-Europe GmbH, Dinslaken, Germany). The binary mobile phase consisted of methanol (solvent A) and methyl tert-butyl ether (MTBE) (solvent B). The solvent gradient was as follows: 0–5 min, 95% A, 1 mL/min; 25 min, 72% A, 1.25 mL/min; 33 min, 5% A, 1.25 mL/min; 40–60 min, 95% A, 1 mL/min.

The absorbance was measured at the wavelength of 450 nm. Compounds were identified based on retention times of commercially available standards (Sigma-Aldrich, Poznań, Poland).

### Determination of total phenolic compounds

Phenolic compounds were obtained by fourfold methanolic (80%, v/v) extraction. Their content was determined spectrophotometrically according to the method described by Dąbrowski et al. [4]. The 0.25 mL of Folin–Ciocalteu reagent solution was added to the dried extract, and 1.5 mL of 14% (m/v) sodium carbonate water solution and 3.25 mL of deionized water were added. After 1 h, absorbance of reaction mixtures was measured at 720 nm against a blank sample using an UV-8000S type spectrophotometer (Shanghai Metash Instruments, Shanghai, China). The phenolic compounds content was calculated on the basis of the d-catechin (Sigma-Aldrich, Poznań, Poland) calibration curve.

### Recovery of phytochemicals

Oil extracted by 5 h with the use of SC-CO_2_ enriched with 10% acetone was recognized as a model oil for calculation of phytochemical recovery. It was calculated by the following Eq. (2):

2 $$\text{Phytochemical}\,\text{recovery}\, ( \text{\%}) = \frac{OR_{sample} \times PC_{sample} }{OR_{model} \times PC_{model} } \times 100\%$$

where OR is the oil recovery (%), and PC is phytochemical content (mg/kg of oil).

### Statistical methods

Statistical analysis of the results was performed using STATISTICA version 12.5 (StatSoft, Kraków, Poland). Principal components analysis (PCA), Pearson correlative coefficients and analysis of variance (ANOVA) with Duncan’s test for homogenous groups (all analyses at *p* ≤ 0.05) were made.

## Results and discussion

### Chia seed oil recovery using various extraction variants

In first stage of the study, the total chia seed lipids were extracted using the Folch method [22]. This procedure uses a mixture of chloroform/methanol (2:1) to tissue disruption which facilitates the release of bound lipids from various cellular compartments [24]. It is recognized as one of the most effective for the extraction of a broad range of lipid classes. The results of this procedure were considered to compare the effectiveness of the SFE variants used.

Oil recovery by the SFE process varied from 27.3% (1 h with pure SC-CO_2_) to 97.4% (5 h with SC-CO_2_ enriched by 10% of acetone) in relation to the total lipid content (Table 1). The 5-h process obtained almost all available lipids (94.1–97.4%), while the 1 h process obtained a maximum of 63.9%. An increase in the extraction time from 1 to 5 h for pure SC-CO_2_ resulted in an approx. 3.4-fold higher yield of oil. Acetone addition favoured oil extractivity and diminished the time-effect. The difference in oil yield between the processes conducted by 1 and 5 h with solvent enriched by 10% of acetone was only 1.5-fold. This significant improvement in extraction rate was caused by easier release of solutes from plant matrices [11]. In this case, the mass transfer resistance is decreased [25]. The higher extractivity of acetone is due to its higher polarity, which enhances the solvating power of SC-CO_2_ for compounds with an amphiphilic character [9].

**Table 1:** Effect of SFE variant on oil recovery from chia seeds and fatty acid composition of the chia seed oils

Extraction time (h)	Acetone addition (%)	Oil recovery (%)	Fatty acids composition (%)								n-6/n-3 ratio (–)
			Palmitic (C16:0)	Stearic (C18:0)	Oleic (C18:1, n-9)	Linoleic (C18:2, n-6)	α-Linolenic (C18:3, n-3)	SFA	MUFA	PUFA	
1	10	63.9	8.7 ± 0.3ab	3.2 ± 0.2a	6.9 ± 0.1bc	21.6 ± 0.0a	59.7 ± 0.5ab	11.9 ± 0.5a	6.9 ± 0.1bc	81.2 ± 0.6b	0.4 ± 0.0a
	6	58.2	9.3 ± 1.4ab	3.2 ± 0.5a	6.7 ± 0.0bc	21.5 ± 0.5a	59.4 ± 1.5ab	12.5 ± 1.9ab	6.7 ± 0.0bc	80.8 ± 2.0ab	0.4 ± 0.0a
	2	43.5	9.4 ± 0.1ab	2.7 ± 0.0a	6.1 ± 0.4a	20.8 ± 0.9a	61.0 ± 1.2b	12.1 ± 0.1ab	6.1 ± 0.4a	81.8 ± 0.2b	0.3 ± 0.0a
	0	27.3	8.8 ± 0.1ab	2.6 ± 0.0a	6.4 ± 0.0b	21.7 ± 0.1a	60.5 ± 0.0b	11.5 ± 0.1a	6.4 ± 0.0ab	82.2 ± 0.1b	0.4 ± 0.0a
5	10	97.4	8.6 ± 0.1a	3.1 ± 0.1a	6.7 ± 0.2bc	21.4 ± 0.4a	60.2 ± 0.4b	11.7 ± 0.2a	6.7 ± 0.2bc	81.6 ± 0.0b	0.4 ± 0.0a
	6	95.7	8.4 ± 0.1a	3.2 ± 0.0a	6.9 ± 0.1c	21.6 ± 0.2a	59.9 ± 0.4ab	11.6 ± 0.1a	6.9 ± 0.1c	81.5 ± 0.2b	0.4 ± 0.0a
	2	94.9	10.6 ± 1.6b	4.0 ± 0.6b	6.9 ± 0.3bc	20.5 ± 1.5a	58.0 ± 0.4a	14.6 ± 2.2b	6.9 ± 0.3bc	78.5 ± 1.9a	0.4 ± 0.0a
	0	94.1	8.6 ± 0.1a	2.9 ± 0.3a	6.3 ± 0.4ab	20.8 ± 0.4a	61.4 ± 1.3b	11.5 ± 0.5a	6.3 ± 0.4ab	82.2 ± 0.8b	0.3 ± 0.0a
Mean		71.9	9.1	3.1	6.6	21.2	60	12.2	6.6	81.2	0.4
SD		27.5	0.7	0.4	0.3	0.5	1.1	1.1	0.3	1.2	0
CV		38.2	7.9	13.7	4.7	2.1	1.8	8.6	4.7	1.5	2.5

Values are mean ± SD (n = 3)

*SD* standard deviation, *CV* coefficient of variance

a, b, …: Means in the same column for all variants followed by different letters are significantly different (*p* ≤ 0.05)

### Fatty acid composition

Only small differences in the fatty acid composition were found between various variants of chia oils (Table 1). Oils were mostly abundant in α-linolenic acid (57.9–61.4%), while other fatty acids were in the following order of abundance: linoleic > palmitic > oleic > stearic (ca. 21, 9, 7 and 3% of the total fraction, respectively). In summary, PUFA, MUFA, and SFA acids accounted for 78.5–82.2, 6.1–6.9, and 11.5–14.6% of the total fraction, respectively. The ratio of n-6/n-3 fatty acids was also almost constant (0.34–0.36). These results agree with the chia fatty acid composition determined by Zanqui et al. [24] and Dąbrowski et al. [4]. In contrast, Ayerza [1] and Uribe et al. [26] found approx. 2–3% lower share of palmitic and linoleic acids, accompanied by a higher share of α-linolenic acid. Determined in present study ratio of n-6/n-3 fatty acids was significantly higher than range of 0.24–0.31 cited by Ixtaina et al. [27] and da Silva Marineli et al. [28]. This ratio can be affected both by seed origin and method of oil extraction. Its lower value is better from nutritional point of view and was determined for example for oil from seeds cultivated in Guatemala than in Argentina and additionally for oil extracted by hexane than obtained by pressing [27].

### Phytosterols

The phytosterol content varied from 4093 to 5060 mg/kg of oil (Table 2). These extreme values were determined in oils extracted by 1 h, for SC-CO_2_ with 10% acetone and pure SC-CO_2_, respectively. This shows that phytosterols are easily extractable by the first portions of supercritical CO_2_. For extraction conducted by 5 h variation of phytosterols, the content between obtained oils was negligible and all oils contained ca. 4662 mg/kg of these compounds. The main representative of phytosterols was β-sitosterol, with a share from 65.2 to 68.9%. Less abundant were campesterol (14.3–16.4%), 25-hydroxy-24-methylcholesterol (8.6–12.1%) and stigmasterol (5.4–7.4%). Comparison of the recovery of phytosterol from seeds showed that from 30.3% (pure SC-CO_2_) to 57.3% (SC-CO_2_ + 10% acetone) of these compounds were extracted in the first hour of the process, while after 5 h phytosterol recovery was at least 95.3%. Among the determined homologues, the share of 25-hydroxy-24-methylcholesterol increased with a growing addition of acetone, and this impact was especially visible in the short-term process (increase from 8.6 to 11.8%). This indicates that the extractivity of more polar phytosterols is facilitated by acetone presence in a solvent stream. Furthermore, the obtained results suggest that at the initial stage of supercritical extraction, phytosterol extractivity is higher than extractivity of other oil compounds. This confirms the results obtained by Sajfrtová et al. [29] for sea-buckthorn seed oil. The cited authors stated that to maximize the concentration of β-sitosterol, the SC-CO_2_ extraction should be stopped after passing over approximately 50 g CO_2_/g of extracted material, when the extraction of β-sitosterol is almost complete. Running the process after reaching this point results in dilution by compounds which require longer extraction.

**Table 2:** Phytosterols content (mg/kg) in chia seed oils obtained by different SFE variants

Extraction time (h)	Acetone addition (%)	Campesterol	Stigmasterol	β-Sitosterol	25-Hydroxy-24-methylcholesterol	Total	Recovery (%)
1	10	670.8 ± 27.8a	271.3 ± 11.9b	2669.0 ± 52.6a	482.3 ± 15.9b	4093.4 ± 2.9a	57.3
	6	684.9 ± 23.9ab	303.3 ± 8.1c	2930.0 ± 91.2b	454.9 ± 15.4a	4373.0 ± 43.8b	55.8
	2	709.0 ± 9.1b	332.7 ± 5.7d	3200.3 ± 62.4c	430.1 ± 1.7a	4672.1 ± 46.0c	44.5
	0	761.8 ± 3.8c	374.6 ± 2.9e	3487.8 ± 67.3d	435.7 ± 7.8a	5059.8 ± 74.3d	30.3
5	10	676.6 ± 2.0ab	252.8 ± 4.1a	3189.8 ± 81.5c	562.8 ± 4.3d	4682.0 ± 79.3c	100.0
	6	683.8 ± 8.8ab	259.1 ± 2.2ab	3195.1 ± 20.8c	568.4 ± 16.7d	4706.4 ± 2.5c	97.9
	2	676.9 ± 8.7ab	255.5 ± 6.6a	3149.0 ± 110.5c	558.8 ± 13.3d	4640.3 ± 81.9c	97.3
	0	658.9 ± 3.4a	257.1 ± 2.7b	3184.1 ± 18.3c	521.3 ± 0.4c	4621.4 ± 12.6c	95.3
Mean		690.3	288.3	3125.6	501.8	4606.0	72.3
SD		32.2	44.9	238.0	58.4	278.9	28.3
CV		4.7	15.6	7.6	11.6	6.1	39.2

Values are mean ± SD (n = 3)

*SD* standard deviation, *CV* coefficient of variance

a, b, …: Means in the same column for all variants followed by different letters are significantly different (*p* ≤ 0.05)

The content of phytosterols in model oil was higher than those determined by Ciftci et al. [2] and Zanqui et al. [24], but extremely lower than that obtained by Álvarez-Chávez et al. [30], who stated that the sterol content in chia oil may vary from 8150 to 12,600 mg/kg. The highest and the lowest values were found in oils extracted from seeds cultivated in Mexico [30] and Brazil [24], respectively. It shows that agri-climate conditions of cultivation highly affect phytosterols content in seeds.

### Tocopherols

The tocopherol content varied from 677.0 to 1243.6 mg/kg of oil (Table 3). These values were determined in oils extracted by 5 h + SC-CO_2_ with 10% acetone and by 1 h with pure SC-CO_2_, respectively. These results show that tocopherol extraction was the fastest at the beginning of the process and that prolongation of time and the addition of acetone leads to dilution of their concentration (a similar mechanism as observed for phytosterols). The impact of acetone addition was especially visible in the case of a short-term process. Recovery of tocopherols was from 51.5 to 74.6% after 1 h of extraction and at least 98.6% for 5 h process. A strong negative correlation (r = − 0.94) between oil recovery and tocopherol content was found (data not shown). According to de Lucas et al. [31], the extraction of tocopherols is faster in the first stages of SFE process and it is the reason for a higher concentration of tocopherols in oil extracted in a shorter time.

**Table 3:** Tocopherols content (mg/kg) in chia seed oils obtained by different SFE variants

Extraction time (h)	Acetone addition (%)	α-	β + γ-	δ-	Total	Recovery (%)
1	10	133.9 ± 5.7b	612.8 ± 3.4c	23.8 ± 0.4a	770.4 ± 8.7c	74.6
	6	100.2 ± 3.9a	723.7 ± 9.5d	34.7 ± 1.6b	858.7 ± 7.3d	75.8
	2	190.7 ± 1.6e	878.1 ± 20.3e	37.9 ± 5.7b	1106.6 ± 16.3e	72.9
	0	210.7 ± 4.7f	984.7 ± 16.8f	48.2 ± 2.5c	1243.6 ± 24.0f	51.5
5	10	141.2 ± 1.2bc	511.7 ± 1.2b	24.2 ± 0.6a	677.0 ± 1.8a	100.0
	6	150.5 ± 2.6d	512.3 ± 2.1b	22.3 ± 0.4a	685.1 ± 0.9a	98.6
	2	146.5 ± 5.8d	521.6 ± 19.1b	27.1 ± 4.1a	695.2 ± 17.4ab	100.8
	0	214.9 ± 0.8f	479.7 ± 1.6a	25.9 ± 1.4a	720.5 ± 3.8b	102.8
Mean		161.1	653.1	30.5	844.6	84.6
SD		40.3	190.6	9.0	215.4	18.6
CV		25.0	29.2	29.6	25.5	22.0

Values are mean ± SD (n = 3)

*SD* standard deviation, *CV* coefficient of variance

a, b, …: Means in the same column for all variants followed by different letters are significantly different (*p* ≤ 0.05)

The main tocopherols were a mixture of β + γ-tocopherol, followed by α- and δ-tocopherols, with an average share of 76.8, 19.6, and 3.6%, respectively. During the first hour of the SFE process, independent of acetone addition, extraction of these homologues was parallel, while prolongation of the SFE time to 5 h facilitated the extraction of α-tocopherol. This effect was mostly visible in oil extracted with the use of pure SC-CO_2_, in which this homologue constituted ca. 30% of total tocopherols (at the same time γ-tocopherol share was diminished). It confirms that α-tocopherol with three –CH_3_ groups is more hydrophobic than β- and γ-tocopherol with two –CH_3_ groups and one –H on the chromanol ring.

It is worth nothing that although the tocopherol content in model chia oil was the lowest, it was still higher than cited in previous studies. Ixtaina et al. [32] and Ciftci et al. [2] found that the range of these compounds was close to 450–500 mg/kg, while Zanqui et al. [24] found only up to 270 mg/kg. The composition of main homologues was also inconsistent. According to Ixtaina et al. [32] and Ciftci et al. [2], the share of γ-tocopherol is close to 95%, while Zanqui et al. [24] found β-tocopherol as the predominant homologue. This inconsistency may result from possible overlapping of these homologues in low resolution and detection effectiveness of chromatographic systems.

### Squalene

Squalene content varied from 18.7 mg/kg (5 h extraction with pure SC-CO_2_) to 63.5 mg/kg (1 h extraction with pure SC-CO_2_) (Table 4). The largest amounts of this compound were found in oils extracted in the short-term process. However, during this short-term process, an increase in acetone modifier addition resulted in an approx. twofold diminished concentration of squalene. Prolongation of extraction time to 5 h caused dilution of squalene by other compounds, so its final concentration in oils did not exceed 22.4 mg/kg. At least 83.7% of this hydrocarbon was extracted with pure SC-CO_2_ in the first hour of the process and practically did not change during the next 4 h of extraction.

**Table 4:** Squalene and polyphenols content in chia seed oils obtained by different SFE variants

Extraction time (h)	Acetone addition (%)	Squalene (mg/kg)	Recovery (%)	Polyphenols (mg/kg)	Recovery (%)
1	10	30.3 ± 0.6c	93.4	33.0 ± 0.8e	80.5
	6	36.9 ± 1.5d	103.6	24.4 ± 1.7bcd	54.4
	2	48.2 ± 1.7e	100.9	21.4 ± 1.0b	35.5
	0	63.5 ± 2.3f	83.7	12.3 ± 1.3a	12.8
5	10	21.3 ± 0.2ab	100.0	26.8 ± 0.4d	100.0
	6	22.4 ± 0.5b	102.3	26.3 ± 1.6 cd	95.6
	2	20.3 ± 0.4ab	93.6	22.7 ± 3.6bc	82.9
	0	18.7 ± 0.7a	84.9	8.9 ± 0.1a	32.1
Mean		32.7	95.3	22.0	61.7
SD		16.0	7.8	7.9	32.6
CV		49.0	8.1	35.9	52.7

Values are mean ± SD (n = 3)

*SD* standard deviation, *CV* coefficient of variance

a, b, …: Means in the same column for all variants followed by different letters are significantly different (*p* ≤ 0.05)

The mechanism of squalene extraction rate was as previously discussed for tocopherols. Lau et al. [33] concluded that squalene extractivity with pure SC-CO_2_ is the best at the initial stage of the process. It is due to the non-polar characteristics of squalene and smaller molecular size than other terpenoid compounds. Similarly, He et al. [34] found the highest squalene concentration in SC-CO_2_-extracted oil from amaranth seeds obtained in conditions that caused a low yield of oil. The strong negative correlation (r = − 0.97) between oil recovery and squalene content determined in the current study (data not shown) confirms this relationship.

References to squalene content in chia oils/seeds are scarce. According to Álvarez-Chávez et al. [30], the amount of squalene in the chia seeds is less than 0.5 g/kg. In the authors’ previous study [4], the content of squalene in chia oils varied from 58.0 to 98.9 mg/kg, depending on the method of oil extraction. The highest values were obtained for SC-CO_2_ and Soxhlet (with acetone)-extracted samples, while the lowest were in *n*-hexane extracted and hot pressed. In contrast, no significant level of this hydrocarbon was detected in cold-pressed chia oil by Bodoira et al. [35].

### Carotenoids

Carotenoid contents varied from 2.28 mg/kg (1 h with pure SC-CO_2_) to 6.21 (1 h with SC-CO_2_ with 10% acetone) (Table 5). The general trend to increase carotenoid contents with an increase of acetone addition was found, and this phenomenon was especially visible in short-term processes (in this variant of extraction, the recovery of carotenoids increased from 12.92 to 82.23%). This confirms the favourable impact of acetone, previously found by Dąbrowski et al. [4]. Prolongation of extraction time was mostly important for pure SC-CO_2_ solvent and, for these variants, an increase in time from 1 to 5 h resulted in an increase of carotenoid recovery from 12.92 to 53.45%.

**Table 5:** Carotenoids content (mg/kg) in chia seed oils obtained by different SFE variants

Extraction time (h)	Acetone addition (%)	Lutein	β-Carotene	9-Cis-β-carotene	Total	Recovery (%)
1	10	3.70 ± 0.24b	2.04 ± 0.13e	0.47 ± 0.04bc	6.21 ± 0.07e	82.23
	6	2.31 ± 0.06a	1.10 ± 0.01c	0.00 ± 0.00a	3.41 ± 0.06c	41.16
	2	1.96 ± 0.36a	0.63 ± 0.10ab	0.00 ± 0.00a	2.59 ± 0.26ab	23.34
	0	1.74 ± 0.47a	0.55 ± 0.06a	0.00 ± 0.00a	2.28 ± 0.41a	12.92
5	10	2.23 ± 0.07a	2.16 ± 0.05ef	0.57 ± 0.07c	4.95 ± 0.05d	100.00
	6	2.11 ± 0.01a	2.23 ± 0.00f	0.46 ± 0.09b	4.80 ± 0.08d	94.44
	2	2.07 ± 0.11a	1.48 ± 0.04d	0.00 ± 0.00a	3.54 ± 0.14c	70.24
	0	1.99 ± 0.03a	0.75 ± 0.11b	0.00 ± 0.00a	2.74 ± 0.08b	53.45
Mean		2.26	1.37	0.19	3.82	59.72
SD		0.61	0.71	0.26	1.38	32.39
CV		26.76	51.72	139.10	36.06	54.23

Values are mean ± SD (n = 3)

*SD* standard deviation, *CV* coefficient of variance

a, b, …: Means in the same column for all variants followed by different letters are significantly different (*p* ≤ 0.05)

The main representative of carotenoids in all oils was lutein (with average share 62.4%), followed by β-carotene (34.1%) and 9-cis-β-carotene (3.6%). However, shares of these compounds were highly related to SFE conditions. Generally, the lowest share of lutein (45.1%) was found in oil extracted by 5 h with the use of 10% acetone enriched SC-CO_2_. In contrast, the highest share (76.3%) of this compound was determined in oil extracted by 1 h with pure SC-CO_2_. The share of β-carotene increased with acetone addition, while 9-cis-β-carotene was only found in oils extracted using a solvent enriched by at least 6 and 10% of acetone for 5 h and 1 h, respectively. These results at first view are unexpected, since lutein (xanthophyll) with two hydroxyl groups should be theoretically better extracted by more polar solvent. However, in plant tissues, lutein primarily exists as an ester with one or two fatty acids [36] and in this form, can probably be more readily extracted/soluble in SC-CO_2_ solvent. For β-carotene, its higher molecular mass causes smaller solubility in SC-CO_2_ and to enhance its solubility, 5–10% of polar modifier (like ethanol) may be utilized [37].

References to carotenoids content in chia oils are scarce. Previous studies showed that the content of carotenoids in chia oil can vary from ca. 1.2 mg/kg [27] to 11.6 mg/kg [38]. These opposite values were found for oils obtained from seeds cultivated in Mexico and Italy, respectively. Method of oil extraction can also affect concentration of these compounds. Dąbrowski et al. [4] using the same batch of seeds but various techniques of oil extraction obtained oils with carotenoid content from 4.1 to 8.4 mg/kg.

### Phenolic compounds

Polyphenol contents varied from 8.9 to 33.0 mg/kg of chia oil (Table 4). The lowest levels were found in both oils extracted by pure SC-CO_2_. The addition of acetone gradually increased the polyphenol concentration in oil and this effect was mostly visible in oil obtained in a short-term process. Recovery of these compounds was highly dependent on the use of acetone as a solvent modifier. For example, oil obtained after 1 h extraction with 2% acetone enriched SC-CO_2_ was similar in this regards to oil obtained after 5 h extraction with pure SC-CO_2_.

The polyphenol content in chia seeds is approx. 940 mg/kg [28]. Only part of it goes to oil during extraction and is composed mostly by chlorogenic acid [27]. According to the method of extraction, chia oils may contain from 0 to 172.4 mg/kg of these compounds [4, 34, 35]. The results of the authors’ previous study [4] showed that chia seed polyphenol extraction is favoured by processes conducted at higher temperatures as well as by the use of more polar solvents. Although detailed analysis of chia polyphenols is scarce, the main groups in plant tissues are flavonoids and phenolic acids. The latter group of compounds is usually esterified to lignocellulose matrix and is generally poorly susceptible to extraction [39]. The relatively high polarity of polyphenols renders extraction with low-polar pure SC-CO_2_ ineffective [40].

### Differentiation of obtained chia oils

**Fig. 1:**
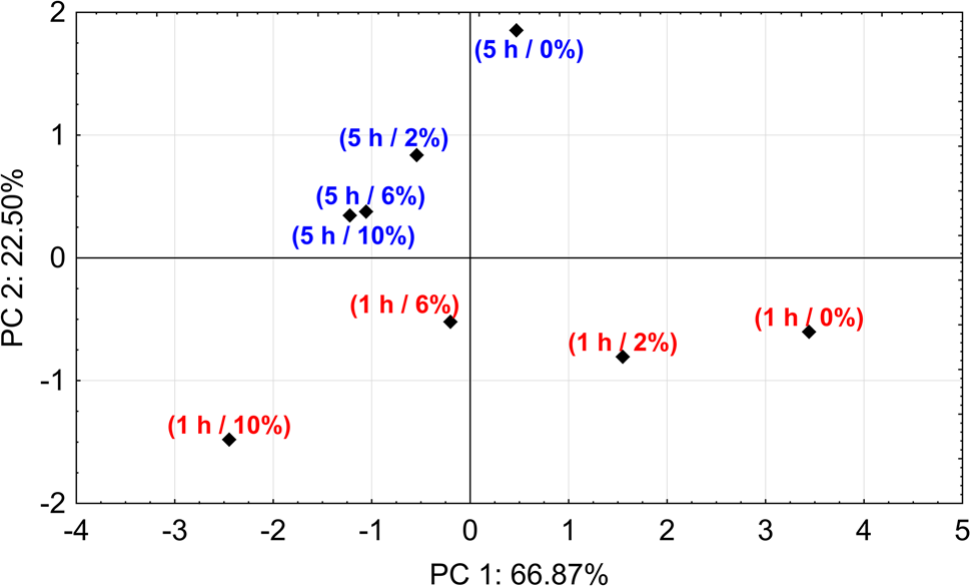
Score plot of the two first principal components after PCA analysis of phytochemicals content in chia seed oils obtained by different extraction variants (extraction time/acetone addition)

PCA (Fig. 1) confirmed the clear differences between the obtained oils. Among them, three oils were the most different: oil extracted 1 h with pure SC-CO_2_ (the most abundant in squalene, sterols, and tocopherols), oil extracted 5 h with pure SC-CO_2_ (poor in squalene, polyphenols, and carotenoids, with a medium level of sterols and tocopherols) and oil extracted 1 h with SC-CO_2_ enriched by 10% acetone (the most abundant in polyphenols and carotenoids). In general, increased polarity of solvent maximized the content of amphiphilic compounds such as polyphenols, while shorter extraction time increased the concentration of the most apolar phytochemicals, such as tocopherols, squalene and phytosterols. Figure 1 also shows that for 1 h extraction each increase of acetone addition from 2 to 10% resulted in shift and differentiation of individual oils. In the case of 5 h extraction dose of acetone addition was less significant, especially for oils extracted with 6 and 10% acetone addition, which were practically identical.

Summarizing the results from Tables 2, 3, 4 and 5 it has been found that the total content of determined phytochemicals varied from 4956 to 6391 mg/kg, and was 79–86% composed of sterols, 12–19% of tocopherols and below 1% of other compounds.
